# Na^+^ Regulation in the Malaria Parasite *Plasmodiumfalciparum* Involves the Cation ATPase PfATP4 and Is a Target of the Spiroindolone Antimalarials

**DOI:** 10.1016/j.chom.2012.12.006

**Published:** 2013-02-13

**Authors:** Natalie J. Spillman, Richard J.W. Allen, Case W. McNamara, Bryan K.S. Yeung, Elizabeth A. Winzeler, Thierry T. Diagana, Kiaran Kirk

**Affiliations:** 1 Research School of Biology, The Australian National University, Canberra, ACT 0200, Australia; 2 Institute for Molecular Bioscience, University of Queensland, Brisbane, QLD 4072, Australia; 3 Genomics Institute of the Novartis Research Foundation, San Diego, CA 92121, USA; 4 Novartis Institute for Tropical Diseases (NITD), 138670 Singapore, Singapore; 5 University of California, San Diego, School of Medicine, La Jolla, CA 92093, USA

## Abstract

The malaria parasite *Plasmodium falciparum* establishes in the host erythrocyte plasma membrane new permeability pathways that mediate nutrient uptake into the infected cell. These pathways simultaneously allow Na^+^ influx, causing [Na^+^] in the infected erythrocyte cytosol to increase to high levels. The intraerythrocytic parasite itself maintains a low cytosolic [Na^+^] via unknown mechanisms. Here we present evidence that the intraerythrocytic parasite actively extrudes Na^+^ against an inward gradient via PfATP4, a parasite plasma membrane protein with sequence similarities to Na^+^-ATPases of lower eukaryotes. Mutations in PfATP4 confer resistance to a potent class of antimalarials, the spiroindolones. Consistent with this, the spiroindolones cause a profound disruption in parasite Na^+^ homeostasis, which is attenuated in parasites bearing resistance-conferring mutations in PfATP4. The mutant parasites also show some impairment of Na^+^ regulation. Taken together, our results are consistent with PfATP4 being a Na^+^ efflux ATPase and a target of the spiroindolones.

## Graphical abstract

**Graphical Abstract:**

**Figure:**
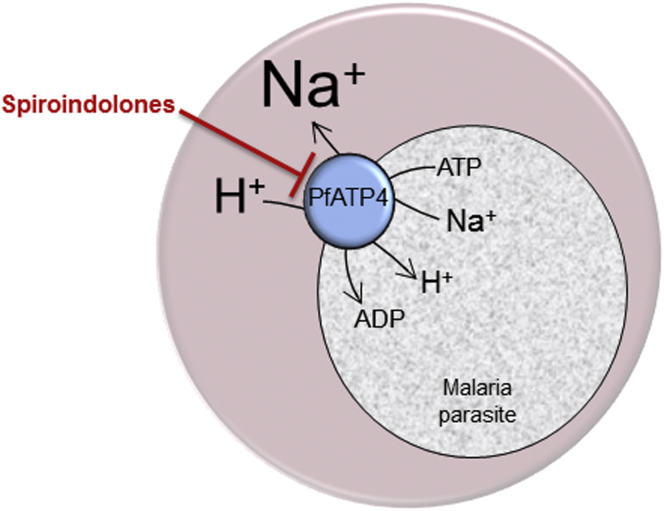

**Highlights:**

► The intraerythrocytic malaria parasite extrudes Na^+^ via a Na^+^-ATPase ► Parasite Na^+^ homeostasis is disrupted by the antimalarial spiroindolones ► Mutations in PfATP4 confer resistance to Na^+^ disruption by the spiroindolones ► PfATP4 is postulated to be a Na^+^ efflux ATPase and a target of the spiroindolones

## Introduction

On entering an uninfected human erythrocyte, an invading *Plasmodium falciparum* malaria parasite passes from the high-[Na^+^]/low-[K^+^] environment of the blood plasma, to the low-[Na^+^]/high-[K^+^] environment of the host cell cytosol (Lee et al., 1988). Some 12–16 hr after invasion, the parasite establishes in the plasma membrane of its host erythrocyte new permeability pathways that mediate the uptake of a range of important nutrients into the infected cell (Martin and Kirk, 2007; Pillai et al., 2012; Saliba et al., 1998) while, at the same time, allowing the influx of Na^+^ and the efflux of K^+^ down their respective concentration gradients. There is a consequent increase in [Na^+^] and decrease in [K^+^] in the erythrocyte cytosol, with both eventually reaching levels approaching those in the extraerythrocytic plasma (Lew et al., 2003; Staines et al., 2001).

Despite the increased [Na^+^] in its immediate extracellular environment, the intraerythrocytic parasite itself maintains a low cytosolic [Na^+^] (Lee et al., 1988; Mauritz et al., 2011; Wünsch et al., 1998). The mechanism by which it does so is unknown. In lower plants (fungi and bryophytes) and some protozoa, Na^+^ extrusion is mediated by an ENA (*e*xitus *na*trus) P-type Na^+^-ATPase (reviewed by Rodríguez-Navarro and Benito, 2010). The ENA ATPases are closely related to sarcoplasmic/endoplasmic reticulum Ca^2+^-ATPases (SERCA) and plasma membrane Ca^2+^-ATPases (PMCA), leading some Na^+^-ATPases within this family to be misannotated as Ca^2+^-ATPases (Benito et al., 2002). The *P. falciparum* genome encodes 13 P-type ATPase candidates (Martin et al., 2005). Two have been annotated as putative Ca^2+^-ATPases; none have been specifically annotated as a Na^+^-ATPase (Martin et al., 2005).

The spiroindolones (Yeung et al., 2010) are a promising class of antimalarials that show low nanomolar activity against blood-stage *P. falciparum* and *P. vivax* parasites (Rottmann et al., 2010). One of the spiroindolones, NITD609, is currently in Phase IIa clinical trials and is “the first molecule with a novel mechanism of action to enter Phase IIa studies for malaria in the last 20 years,” (http://www.mmv.org/research-development/rd-portfolio). In *P. falciparum*, mutations in PfATP4, a P-type ATPase candidate on the parasite plasma membrane (encoded by PF3D7_1211900, previously PFL0590c), confer resistance to the spiroindolones (Rottmann et al., 2010). PfATP4 has been annotated, on the basis of sequence homology, as a Ca^2+^-transporting ATPase. There is evidence for it having an associated Ca^2+^-dependent ATPase activity (Krishna et al., 2001); however, this has not been confirmed (Rottmann et al., 2010), and a Ca^2+^ transport function has not been demonstrated directly.

The aim of this study was to investigate the mechanism(s) of Na^+^ regulation in *P. falciparum* and to characterize the transporter(s) involved. The results are consistent with the hypothesis that PfATP4 is a plasma membrane Na^+^ efflux pump, similar to the ENA Na^+^-ATPases of other lower eukaryotes, and a target of the spiroindolone class of antimalarials.

## Results

### Na^+^ Regulation in the Intraerythrocytic Parasite Involves a P-type ATPase

To investigate Na^+^ regulation in the parasite, mature *P. falciparum* 3D7 trophozoites were functionally isolated from their host erythrocytes by saponin permeabilization of the host cell membrane and loaded with the fluorescent Na^+^-sensitive dye SBFI. Fluorescence was distributed uniformly throughout the cytosol of the dye-loaded parasites (Figure S1A), and the fluorescence ratio increased with increasing intracellular [Na^+^] ([Na^+^]_i_), allowing calibration of the method (Figures S1B and S1C). For parasites suspended at an extracellular [Na^+^] ([Na^+^]_o_) of 125 mM, the resting [Na^+^]_i_ was estimated to be 11.0 ± 0.6 mM (mean ± SEM, n = 34). Upon replacement of extracellular Na^+^ with an alternative cation (choline^+^, N-methyl-D-glucamine^+^, or K^+^), [Na^+^]_i_ decreased to close to zero within a few minutes (Figure S1D). Conversely, on increasing [Na^+^]_o_, [Na^+^]_i_ underwent a modest increase. When [Na^+^]_o_ was increased by 175 mM to 300 mM, more than double the physiological level, the increase in [Na^+^]_i_ (after correction for osmotic cell shrinkage) was less than 20 mM (Figures S1E and S1F). The parasite is therefore capable of maintaining a low [Na^+^]_i_ even when exposed to a very high [Na^+^]_o_.

**Figure 1:**
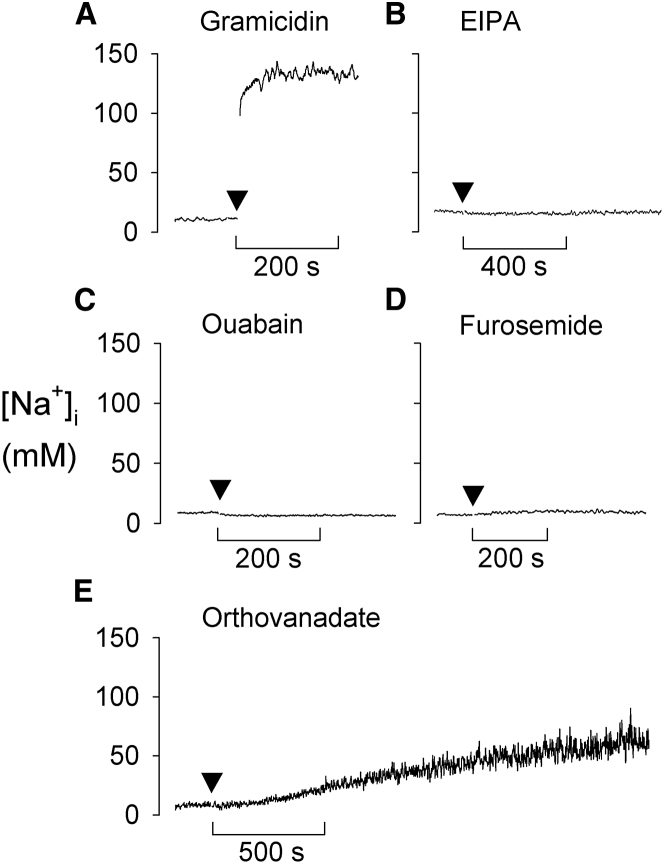
Effects of an Ionophore and Ion Transport Inhibitors on [Na^+^]_i_ in Saponin-Isolated, SBFI-loaded *P. falciparum* Trophozoites (A–E) [Na^+^]_i_ traces showing the effect of addition (at the point indicated by the closed triangle) of (A) gramicidin (5 μM), (B) EIPA (20 μM), (C) ouabain (2 mM), (D) furosemide (100 μM), and (E) orthovanadate (100 μM). For all additions except ouabain, the compounds were added as a concentrated stock. Cells were exposed to 2 mM ouabain by being sedimented by centrifugation then resuspended at the time point indicated in an equivalent saline containing the inhibitor. The traces shown in each case are representative of those obtained from at least three independent cell preparations. See also Figure S1.

To investigate the mechanisms involved in maintaining the low [Na^+^]_i_ in the parasite, various ionophores and ion transport inhibitors were tested for their effect on [Na^+^]_i_. Gramicidin (5 μM), a monovalent-cation selective ionophore, induced a rapid increase in [Na^+^]_i_, with [Na^+^]_i_ approaching [Na^+^]_o_ (125 mM; Figure 1A). Neither the Na^+^/H^+^ exchanger inhibitor ethylisopropylamiloride (EIPA; 20 μM) nor the Na^+^/K^+^-ATPase inhibitor ouabain (2 mM) had a significant effect on resting [Na^+^]_i_ (Figures 1B and 1C). Furosemide (100 μM), an inhibitor of some protozoal Na^+^-ATPases (De Souza et al., 2007a; Iizumi et al., 2006), caused a small (2.3 ± 0.7 mM) increase in [Na^+^]_i_ (n = 4; p = 0.009) (Figure 1D). Sodium orthovanadate (100 μM), a phosphate analog that inhibits P-type ATPases (Cantley et al., 1978a, 1978b), caused [Na^+^]_i_ to undergo a prolonged time-dependent increase (Figure 1E). This is consistent with a P-type ATPase playing a role in the efflux of Na^+^ from the parasite, countering the influx of Na^+^ down its inward electrochemical gradient. The antimalarials chloroquine (10 μM) and artemisinin (10 μM) were both found to have no effect on [Na^+^]_i_ on the timescale of the experiments (Figures S1G and S1H).

Suspension of isolated parasites in glucose-free medium (conditions under which parasites undergo ATP depletion; Saliba and Kirk, 1999) resulted in a progressive increase in [Na^+^]_i_ (Figure S1I), again consistent with the involvement of an ATPase in the maintenance of a low resting [Na^+^]_i_.

### Spiroindolones Perturb [Na^+^]_i_ and pH_i_, but Not [Ca^2+^]_i_

**Figure 2:**
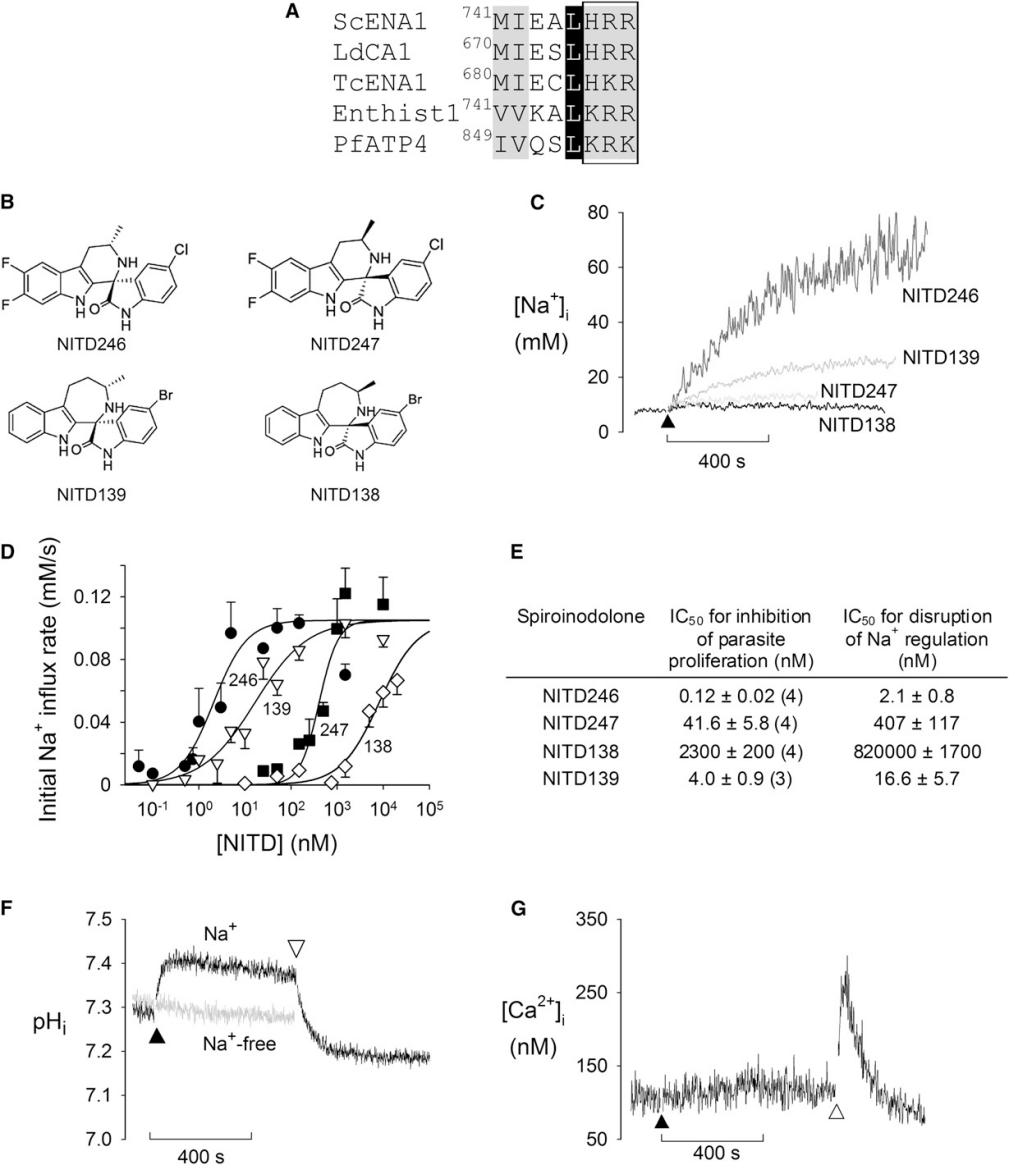
PfATP4 and the Effect of the Spiroindolones on Ion Regulation in Saponin-Isolated *P. falciparum* Trophozoites (A) Amino acid alignment of residues 849–856 of PfATP4 with the equivalent regions in ENA Na^+^-ATPases from *Saccharomyces cerevisiae* (ScENA1; P13587), *Leishmania donovani* (LdCA1; AAC19126), *Trypanosoma cruzi* (TcENA1; XP_817442.1), and *Entamoeba histolytica* (Enthist1; XM_652464). Black shaded residues are completely conserved; gray shaded residues are functionally conserved. The alignment is based on a previous alignment of fungal, bryophyte, and protozoal ENA ATPases from Rodríguez-Navarro and Benito (2010) who highlighted the conservation of this eight amino acid motif (^741^MIEALHRR in ScENA1) in ENA ATPases. The ^854^KRK triple-basic motif in PfATP4 (boxed) plays an important role in Na^+^ transport in ENA Na^+^-ATPases and is absent from PMCA, SERCA, and Na^+^/K^+^-ATPases (Rodríguez-Navarro and Benito, 2010). The triple-basic motif is not present in the same position in any of the other annotated *P. falciparum* P-type ATPases (including PfATPase1 [PFE0805w], PfATPase3 [PFE0195w], PfATP6 [PFA0310c], and two putative cation-transporting P-type ATPases [MAL13P1.246, PF07_0115]). Note that the PfATP4 sequence used (from PlasmoDB: PFL0590c) was the updated sequence reannotated to correct a missed nucleotide, thus removing a previously incorrectly annotated intron. (B) Chemical structures of the enantiomers NITD246/NITD247 and NITD138/NITD139. (C) Traces showing the effects of the four spiroindolones, each at a concentration of 50 nM, on [Na^+^]_i_ in SBFI-loaded 3D7 parasites suspended in standard saline. The spiroindolones were added at the time point indicated by the closed triangle. (D) Concentration dependence of the effect of each of the four spiroindolones on the initial rate of Na^+^ influx (● NITD246; ■ NITD247; ◇ NITD138; ▿NITD139). The initial rate of Na^+^ influx was estimated from traces such as those represented in (C) (see also Figure S2A) as described in Experimental Procedures. Each data point represents the mean Na^+^ influx rate averaged from at least three independent experiments and is shown ±SEM. For the purpose of the curve fitting, the maximum rate of Na^+^ influx (y^max^ in the sigmoidal curve described in Experimental Procedures) was set to 0.11 mM/s, the mean of the Na^+^ influx rates measured using the maximally effective concentrations of the three most potent inhibitors (NITD246, NITD139, and NITD247). (E) Summary of the IC_50_ values for inhibition of parasite proliferation and for disruption of [Na^+^]_i_ regulation (i.e., the concentration of each inhibitor required to cause the [Na^+^]_i_ to increase from its normal resting value at half the maximal rate). The IC_50_ values cited for inhibition of parasite proliferation are the mean ± SEM of those estimated in the number of independent experiments shown in parentheses (with each independent experiment performed in triplicate). The IC_50_ values for disruption of [Na^+^]_i_ regulation are derived from the fitted curves shown in (D). (F) Traces showing the effects on pH_i_ of the addition of NITD246 (25 nM, at the point indicated by the black triangle) followed by the addition of concanamycin A (75 nM, at the point indicated by the open triangle) to BCECF-loaded parasites suspended in either standard saline (black trace) or Na^+^-free solution (in which Na^+^ was replaced with an equimolar concentration of choline^+^; gray trace). See also Figure S2C for a similar trace using orthovanadate instead of NITD246. (G) Trace showing the effect of the addition of NITD246 (50 nM, at the point indicated by the closed triangle) and CPA (2 μM, at the point indicated by the open triangle) on [Ca^2+^]_i_ in fura-2-loaded parasites suspended in standard saline supplemented with 1 μM Ca^2+^. All of the traces shown are, in each case, representative of those obtained from at least three independent cell preparations. See also Figure S2.

Some lower eukaryotes, including other protozoa, use an ENA P-type Na^+^-ATPase to extrude Na^+^ and thereby maintain a low [Na^+^]_i_ (De Souza et al., 2007a; Iizumi et al., 2006; Stiles et al., 2003). Amino acid alignments using the sequences of ENA Na^+^-ATPase family members from *Saccharomyces* (ScENA1; CAA98867; 29% identity with PfATP4), *Leishmania* (LdCA1; AAC19126; 29% identity), *Trypanosoma* (TcENA1; XP_817442.1; 29% identity), and *Entamoeba* (Enthist1; XM_652464; 39% identity) revealed homology between these proteins and PfATP4. In particular, PfATP4 contains an eight amino acid motif (^849^IVQSLKRK) that is highly conserved in ENA Na^+^-ATPases (Figure 2A). Within this motif is a triple-basic amino acid sequence (^854^KRK in PfATP4) that is important for Na^+^ transport in ENA Na^+^-ATPases and absent from both SERCA and PMCA as well as from Na^+^/K^+^-ATPases (Rodríguez-Navarro and Benito, 2010). The presence of this amino acid triplet in PfATP4 lends strong support to the hypothesis that PfATP4 functions as a Na^+^ efflux pump.

Mutations in PfATP4 confer resistance to the spiroindolone antimalarials (Rottmann et al., 2010). We therefore investigated the effect of the spiroindolones on parasite Na^+^ homeostasis. In initial experiments, two enantiomeric pairs of spiroindolones—NITD246/NITD247 and NITD138/NITD139 (Figure 2B), with each compound having an enantiopurity of >98% enantiomeric excess—were tested for their effect on the resting [Na^+^]_i_. Addition of NITD246 and NITD139 (50 nM) resulted in an immediate rapid increase in [Na^+^]_i_ (Figure 2C). Addition of their much less active enantiomers, NITD247 and NITD138, respectively, had little effect at the same concentration (Figure 2C). The less active enantiomer preparations did perturb [Na^+^]_i_ at much higher concentrations, most likely due to the presence of trace amounts of the active enantiomer in the samples. For all four compounds the effect on [Na^+^]_i_ was dose dependent (see Figure S2A for representative traces); for each compound, an IC_50_ (half maximal inhibitory concentration) for [Na^+^]_i_ disruption was calculated based upon the initial rate of increase of [Na^+^]_i_ following spiroindolone addition. The order of potency for the effect of the four compounds on [Na^+^]_i_ was the same as their order of potency for inhibition of parasite proliferation (i.e., NITD246 > NITD139 > NITD247 > NITD138; Figures 2D and 2E). The rates of increase of [Na^+^]_i_ measured at maximally effective concentrations of NITD246, NITD139, and NITD247 (0.092 ± 0.007 mM/s, 0.098 ± 0.007 mM/s, and 0.125 ± 0.013 mM/s, respectively) were not significantly different from one another (p > 0.06), or from the rate of increase of [Na^+^]_i_ seen following the addition of 500 μM orthovanadate (0.13 ± 0.03 mM/s; n = 4, p = 0.57). These data are consistent with each of these compounds inhibiting the parasite’s Na^+^ extrusion mechanism, revealing in each case the endogenous influx of Na^+^ into the parasite. For the least potent spiroindolone, NITD138, the rate of increase of [Na^+^]_i_ did not reach the maximum value at the highest concentration tested, precluding an estimate of a maximum rate of increase of [Na^+^]_i_. None of the tested spiroindolones had any effect on parasite ATP levels when tested at a concentration of 50 nM and measured over a period of 60 min (Figure S2B).

Having shown that the spiroindolones disrupt parasite [Na^+^]_i_ regulation, we went on to assess their specificity by testing the most potent of these compounds, NITD246, for its effect on the cytosolic concentrations of other ions. As shown in Figure 2F, addition of 50 nM NITD246 to isolated parasites suspended in a (Na^+^-containing) medium (pH 7.1) caused the cytosolic pH (pH_i_) to increase from 7.33 ± 0.03 to 7.46 ± 0.02 (n = 6, p = 0.009). The NITD246-induced alkalinisation was Na^+^ dependent; addition of 50 nM NITD246 to parasites washed and resuspended in a solution containing choline^+^ in place of Na^+^ (conditions under which [Na^+^]_i_ was close to zero; Figure S1D) had no significant effect on pH_i_ (Figure 2F). The P-type ATPase inhibitor orthovanadate (100 μM) caused a similar Na^+^-dependent increase in pH_i_ from 7.28 ± 0.03 to 7.36 ± 0.03 (n = 6, p = 0.024; Figure S2C).

An increase in pH_i_, as was seen following the addition of NITD246 or orthovanadate, represents an increase in the transmembrane [H^+^] gradient. Such an increase in a transmembrane ion gradient can only occur through the involvement of an active (i.e., energy-requiring) transport process, such as an ion-pumping ATPase. The primary acid extrusion mechanism in the malaria parasite, responsible for maintaining pH_i_ above the extracellular pH (pH_o_), is a plasma membrane V-type H^+^-ATPase (Hayashi et al., 2000; Saliba and Kirk, 1999; Spillman et al., 2008). The possible involvement of this H^+^ pump in the alkalinisation seen in response to the addition of NITD246 or orthovanadate was investigated using the V-type H^+^-ATPase inhibitor concanamycin A. Upon addition of concanamycin A to parasites that had undergone an NITD246- or orthovanadate-induced alkalinisation, there was an immediate reversal of the alkalinisation, with pH_i_ decreasing to below its normal resting value (Figures 2F and S2C, respectively). The NITD246- and orthovanadate-induced alkalinisation may therefore be attributed to the uninhibited action of the V-type H^+^-ATPase.

Because PfATP4 has previously been annotated as a Ca^2+^-transporting ATPase, we investigated the effect of NITD246 on cytosolic [Ca^2+^] ([Ca^2+^]_i_). In contrast to its effect on [Na^+^]_i_ and pH_i_, the addition of NITD246 (50 nM) to isolated parasites suspended in medium containing 1 μM Ca^2+^ (under which conditions there is an inward Ca^2+^ gradient) had no effect on [Ca^2+^]_i_ (Figure 2G). Under the same conditions, the SERCA Ca^2+^-ATPase inhibitor cyclopiazonic acid (CPA; 2 μM) did cause a transient increase in [Ca^2+^]_i_, as observed previously (Alleva and Kirk, 2001). CPA and another SERCA Ca^2+^-ATPase inhibitor, thapsigargin, were also tested for their effect on [Na^+^]_i_. While thapsigargin (2 μM) had little effect, the addition of CPA (40 μM) caused [Na^+^]_i_ to increase (Figures S2D and S2E).

### The Response of the Parasite to an Imposed Intracellular Na^+^ Load

**Figure 3:**
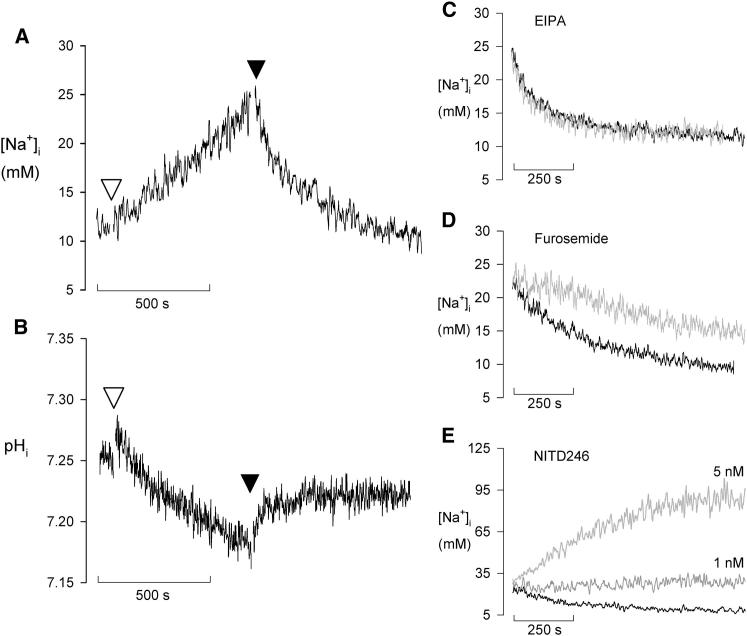
Response of Saponin-Isolated *P. falciparum* Trophozoites to an Imposed Intracellular Na^+^ Load (A) Trace showing the effect of removal of extracellular K^+^ on [Na^+^]_i_ in SBFI-loaded parasites. At the time point indicated by the open triangle, the cells (in standard saline) were washed twice by centrifugation and resuspension in a K^+^-free saline (in which K^+^ was replaced isosmotically with Na^+^). At the time point indicated by the closed triangle, 10 mM K^+^ (as KCl) was added to the suspension. The trace is representative of that obtained from at least twenty independent cell preparations. (B) Trace showing the effect of the same maneuvers (i.e., removal of extracellular K^+^ at the point indicated by the open triangle then restoration at the point indicated by the closed triangle) on the pH_i_ in BCECF-loaded parasite suspension. The trace is representative of that obtained from at least seven independent cell preparations. (C–E) Effect of ion transport inhibitors on the recovery of [Na^+^]_i_ from an imposed intracellular Na^+^ load. SBFI-loaded parasite suspensions were subjected to a Na^+^ load (by suspension in K^+^-free medium) as illustrated in (A), and the traces commenced with the addition to the suspension of 10 mM KCl either with (gray traces) or without (control, black traces) (C) EIPA (20 μM), (D) furosemide (100 μM), or (E) NITD246 (1 nM or 5 nM). The traces shown are each representative of those obtained from at least three independent cell preparations.

As part of this study we investigated whether [Na^+^]_i_ was affected by varying the extracellular concentration of ions other than Na^+^. Strikingly, on removal of K^+^ from the medium (by replacement of the 5 mM K^+^ present in standard saline with an equivalent concentration of Na^+^) there was a progressive increase in [Na^+^]_i_ (Figure 3A). The increase was approximately linear with time, occurring at a rate of 8.8 ± 0.9 × 10^−3^ mM/s (n = 15; i.e., some 10-fold lower than the maximum rate of increase seen in response to the addition of the spiroindolones or orthovanadate). The increase in [Na^+^]_i_ seen on removal of extracellular K^+^ was accompanied by a time-dependent decrease in pH_i_ (0.026 ± 0.003 pH units/min; n = 9; Figure 3B). When K^+^ was restored to the medium by the addition of 10 mM KCl, [Na^+^]_i_ recovered to a level not significantly different from the initial resting [Na^+^]_i_ (recovery to 9.5 ± 2.7 mM; n = 15; p = 0.15), and pH_i_ increased, albeit not quite to the initial resting value. The decrease in [Na^+^]_i_ seen following the restoration of K^+^ to the medium provides a direct demonstration of a net efflux of Na^+^ from the parasite against an inward electrochemical gradient and, therefore, of the presence of an active Na^+^ efflux transporter.

A reduction in the extracellular [K^+^] has previously been shown to result in a hyperpolarization of the parasite plasma membrane (Allen and Kirk, 2004). The mechanism responsible for the increase in [Na^+^]_i_ seen upon removal of extracellular K^+^ was not investigated further here. However, the phenomenon does provide a useful means of imposing an intracellular Na^+^ load on the parasite and of testing the effect of inhibitors on the net efflux of Na^+^ from the Na^+^-loaded parasite. In experiments in which parasites were loaded with additional intracellular Na^+^ by the sequential removal and restoration of extracellular K^+^, the recovery of [Na^+^]_i_ was (1) unaffected by the Na^+^/H^+^ exchanger inhibitor EIPA (20 μM; Figure 3C); (2) slowed by furosemide (100 μM; Figure 3D); and (3) prevented by the spiroindolone NITD246 (at 1 nM, NITD246 resulted in [Na^+^]_i_ remaining at the level reached at the point of addition of extracellular K^+^; at 5 nM, it resulted in a prolonged time-dependent increase in [Na^+^]_i_; [Figure 3E]).

### A Spiroindolone-Sensitive Membrane-Associated Na^+^-ATPase in the Parasite

To test directly whether the spiroindolones inhibit a membrane ATPase in the parasite, we investigated ATPase activity (i.e., ATP hydrolysis) in membrane preparations from both infected and uninfected erythrocytes. The total membrane-associated ATPase activity in erythrocytes infected with mature trophozoite-stage 3D7 parasites (14 ± 3 nmol P_i_ released/min/10^8^ cells; n = 5), measured in the presence of 100 mM Na^+^, was ∼14-fold higher than that in uninfected erythrocytes (1.0 ± 0.2 nmol P_i_ released/min/10^8^ cells; n = 5).

**Figure 4:**
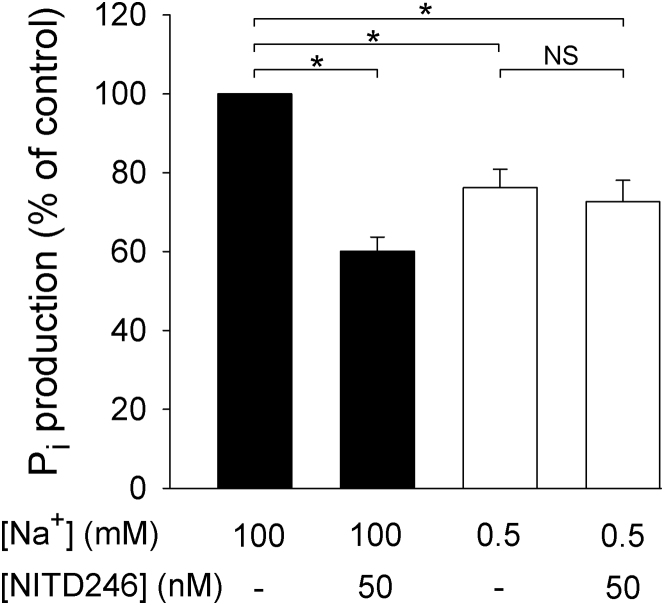
Na^+^-Dependence and Spiroindolone-Sensitivity of Membrane-Associated ATPase Activity in *P. falciparum* Infected Human Erythrocytes ATPase activity was estimated from the rate of production of P_i_ and measured using the PiColorLock Gold Phosphate Detection Kit following the addition of 0.25 mM ATP. Membrane preparations were suspended in either a high (100 mM) Na^+^ solution (black bars) or a low (0.5 mM) Na^+^ solution (in which Na^+^ was replaced with equimolar choline; white bars) in the absence or presence of 50 nM NITD246. ATPase activity is expressed as a percentage of that measured in high-Na^+^ medium in the absence of inhibitor (control). Asterisks indicating a statistically significant difference from the control (p < 0.05); NS denotes p > 0.05. The data are averaged from five independent experiments and are shown +SEM.

On reduction of the [Na^+^] in the reaction buffer to 0.5 mM, the ATPase activity in the infected erythrocyte membrane preparation decreased to 76% ± 5% of the control value (n = 5; p = 0.019, paired t test; Figure 4). A significant fraction of the membrane-associated ATPase activity in parasitized erythrocytes was therefore Na^+^ dependent.

On addition of the spiroindolone NITD246 (50 nM) to infected erythrocyte membranes suspended in the presence of 100 mM Na^+^, the ATPase activity decreased to 60% ± 4% of the control value (n = 5; p = 0.020, paired t test; Figure 4). However, addition of NITD246 (50 nM) to infected erythrocyte membranes suspended in the low (0.5 mM) [Na^+^] medium resulted in no significant change in the ATPase activity (p = 0.63; Figure 4); i.e., the spiroindolone-sensitive ATPase activity was present under high-[Na^+^], but not under low-[Na^+^], conditions. It should be noted that the ATPase activity measured under low-[Na^+^] conditions (both in the presence and absence of NITD246) was higher than that measured under high-[Na^+^] conditions in the presence of NITD246, consistent with the stimulation of one or more additional spiroindolone-insensitive ATPases in the low-[Na^+^] solution.

The spiroindolone had no effect on the (much lower) ATPase activity of membranes from uninfected erythrocytes (data not shown).

### Reduced Spiroindolone Sensitivity of [Na^+^] Regulation and Na^+^-ATPase Activity in Parasites with Mutations in PfATP4

In the original study reporting the antimalarial activity of the spiroindolones (Rottmann et al., 2010), spiroindolone-resistant *P. falciparum* parasites (NITD609-R^Dd2^) were generated by exposing parasites to incrementally increasing (sublethal) concentrations of the potent spiroindolone, NITD609. In all such experiments the spiroindolone-resistant parasites acquired mutations in *pfatp4*, and introduction of these mutations into spiroindolone-sensitive (Dd2^attB^) parasites conferred spiroindolone resistance (Rottmann et al., 2010).

Two spiroindolone-resistant lines expressing mutant PfATP4 (the spiroindolone-exposed NITD609-R^Dd2^ clone #2 and the transfectant Dd2^attB^ CAM I398F/P990R line) generated in the earlier study were used here to investigate the role of PfATP4 in parasite Na^+^ regulation.

**Figure 5:**
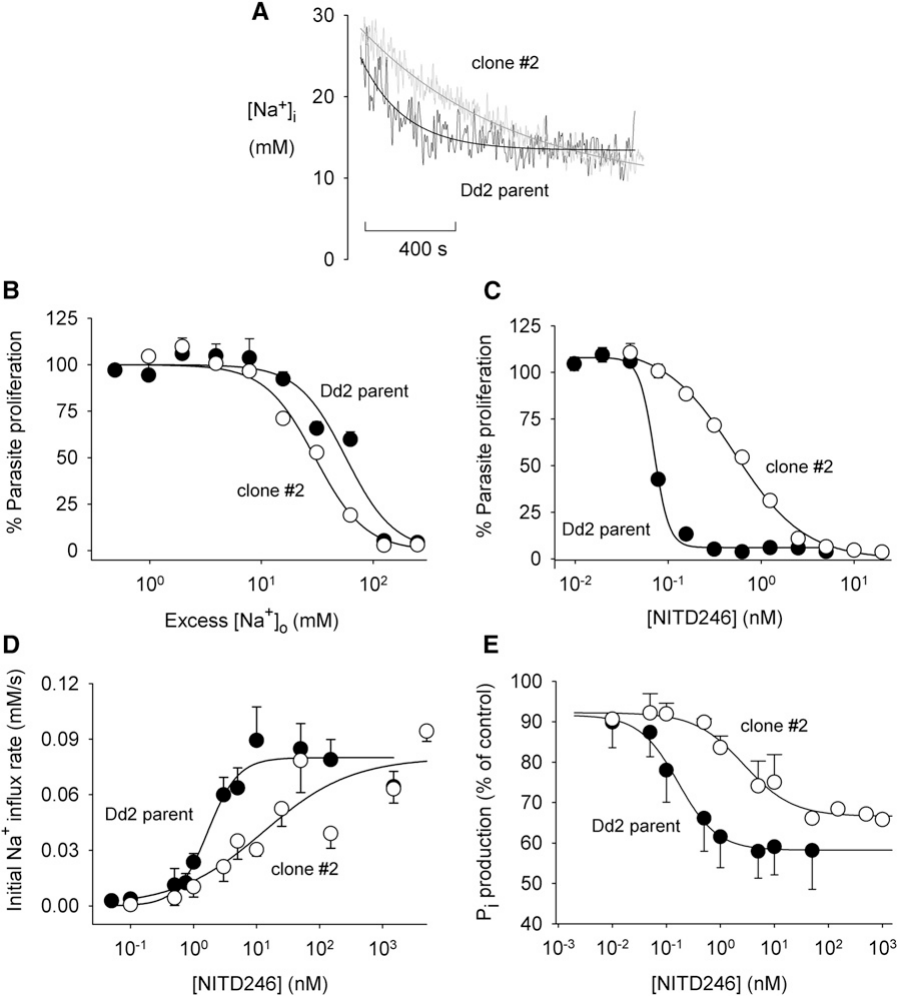
Comparisons of PfATP4 NITD609-R^Dd2^ Clone #2 Parasites with their Dd2 Parent Line. The PfATP4 NITD609-R^Dd2^ Clone #2 Parasites were Generated by Exposure of Dd2 *P. falciparum* Parasites to the Potent Spiroindolone NITD609 (A) Recovery of [Na^+^]_i_ following an intracellular Na^+^ load imposed by the removal and restoration of extracellular K^+^ (as illustrated in Figure 3). The traces are representative of those obtained from at least three independent cell preparations, and the smooth lines are the fitted curves (see Experimental Procedures). The grey trace is from PfATP4 NITD609-RDd2 clone #2 parasites, and the black trace is from the Dd2 parent line. (B) Inhibition of parasite proliferation by excess extracellular Na^+^, with “Excess [Na^+^]_o_” denoting the increase in [Na^+^]_o_ above that normally present in the parasite culture medium (∼133 mM). (C) Inhibition of parasite proliferation by NITD246. (D) Disruption of parasite [Na^+^] regulation by NITD246 (estimated from the rate of increase of the [Na^+^]_i_ immediately following the addition of NITD246 to isolated SBFI-loaded parasites). (E) Inhibition of membrane-associated ATPase activity by NITD246 (measured as the rate of ATP hydrolysis in membranes isolated from parasitized erythrocytes). In (B)–(E) the data are averaged from at least three experiments and are shown ±SEM. In (B)–(E) the open symbols are from PfATP4 NITD609-RDd2 clone #2 parasites, and the closed symbols are from the Dd2 parent line. All data in (B)-(E) are averaged from at least three experiments and are shown ±SEM. The corresponding IC_50_ values are given in Table 1.

An initial characterization of the two mutant PfATP4 lines revealed that for both of the two parasite lines expressing mutant PfATP4, there was, relative to the parental line, (1) a small increase in the resting [Na^+^]_i_ (Table 1); (2) a decrease in the rate of efflux of Na^+^ following an imposed Na^+^ load (Table 1 and Figure 5A); and (3) an increased sensitivity to the growth-inhibitory effects of supraphysiological extracellular Na^+^ concentrations (Table 1 and Figure 5B). The parasites expressing mutant PfATP4 therefore showed a slight impairment in their [Na^+^]_i_ regulation.

As expected, the spiroindolone-resistant lines showed a significant decrease in their sensitivity (relative to the parental lines) to growth inhibition by the spiroindolone NITD246 (Table 1 and Figure 5C). They showed a similar decrease in their sensitivity to disruption of [Na^+^]_i_ by NITD246 (Table 1 and Figure 5D) and to inhibition of membrane-associated ATPase activity by NITD246 (Table 1 and Figure 5E).

## Discussion

**Figure 6:**
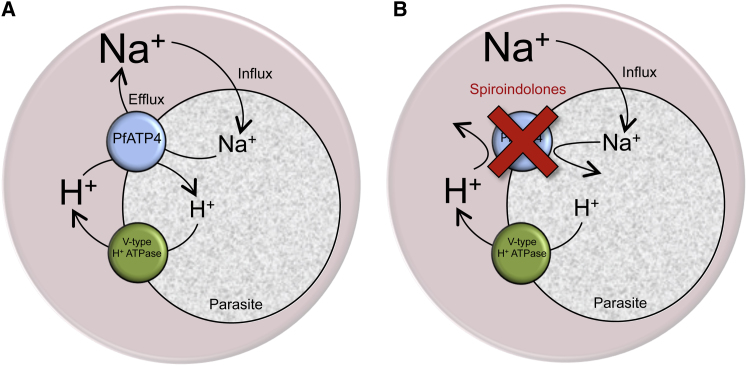
Schematic Representation Showing the Proposed Role of PfATP4 in Na^+^ Homeostasis in the Intraerythrocytic *P. falciparum* Trophozoite-Stage Parasite (A) PfATP4 is postulated to function as an ENA Na^+^-ATPase, actively extruding Na^+^ from the intraerythrocytic parasite, countering the influx of Na^+^ (which occurs via unknown pathways), and maintaining a [Na^+^]_i_ (∼11 mM) more than 10-fold lower than [Na^+^]_o_ (125 mM in the experiments conducted here). The PfATP4-mediated efflux of Na^+^ is postulated to be accompanied by an influx of H^+^ ions, and this constitutes a significant acid load, which is countered by H^+^ extrusion via the parasite’s plasma membrane V-type H^+^-ATPase. (B) PfATP4 is inhibited by the spiroindolones (as well as by orthovanadate and CPA). Inhibition of PfATP4 results in an increase in [Na^+^]_i_ (Figure 2C) as Na^+^ moves into the cell, down its electrochemical gradient, via the Na^+^ influx pathways. At the same time there is an increase in pH_i_ (Figure 2F) attributable to the V-type H^+^-ATPase now operating in the absence of the PfATP4-mediated acid load. The alkalinisation seen following inhibition of PfATP4 is not seen for parasites washed and resuspended in Na^+^-free medium (Figure 2F), as under these conditions [Na^+^]_i_ is close to zero (Figure S1D), PfATP4 is nonfunctional, and there is therefore no PfATP4-mediated acid load.

The findings here that Na^+^ regulation in asexual blood-stage *P. falciparum* parasites was impaired by the P-type ATPase inhibitor orthovanadate, showed some sensitivity to the ENA Na^+^-ATPase inhibitor furosemide, and was unaffected by ouabain (which, at the 2 mM concentration tested here, inhibits all known Na^+^/K^+^-ATPases [De Souza et al., 2007b]), are all consistent with the parasite relying on an ENA Na^+^-ATPase to extrude Na^+^, countering the influx of Na^+^, and thereby maintaining a low [Na^+^]_i_ (represented schematically in Figure 6). Sequence analysis revealed PfATP4 as the most likely candidate for a *P. falciparum* ENA Na^+^-ATPase. The location of PfATP4 on the parasite plasma membrane (Dyer et al., 1996; Rottmann et al., 2010) is consistent with it playing a role in the extrusion of Na^+^ from the parasite, and the finding in this study that mutations in PfATP4 result in altered Na^+^ regulation in the parasite (increased resting [Na^+^]_i_, reduced Na^+^ efflux following an imposed intracellular Na^+^ load, and increased sensitivity to the growth-inhibitory effects of increased extracellular [Na^+^]) provides further support for this hypothesis.

The finding by Rottmann et al. (2010) that mutations in PfATP4 confer resistance to the spiroindolones raises the obvious possibility that PfATP4 is a target of these compounds. The findings here that spiroindolones disrupt parasite Na^+^ regulation with the same order of potency as was seen for inhibition of parasite proliferation (i.e., NITD246 > NITD139 > NITD247 > NITD138) and that the resistance-conferring mutations in PfATP4 confer reduced sensitivity to both the disruption of Na^+^ regulation and the inhibition of membrane-associated ATPase activity by NITD246 are consistent with this hypothesis. The finding that, under conditions in which NITD246 disrupts Na^+^ regulation, it does not induce a rise in [Ca^2+^]_i_ argues against the spiroindolones exerting their effect on parasite growth via an effect on Ca^2+^ regulation. The observation that the SERCA Ca^2+^-ATPase inhibitor CPA caused a disruption of Na^+^ regulation might be accounted for by the CPA binding pocket being conserved in PfATP4, though the possibility that the increase in [Na^+^]_i_ is secondary to a CPA-induced increase in [Ca^2+^]_i_ cannot be excluded.

The transfectant (Dd2^attB^ CAM I398F/P990R) parasite line showed a lower degree of resistance to the growth-inhibitory effects of the spiroindolones than the drug-selected mutant (NITD609-R^Dd2^ clone #2) parasite line (Rottmann et al., 2010 and present study). Consistent with this, the transfectants showed a lower level of resistance than the drug-selected line to both the [Na^+^]_i_-disrupting effect of NITD246 and inhibition by NITD246 of the membrane-associated ATPase activity. While the drug-selected line has a single (mutant) copy of the *pfatp4* gene, the transfectants coexpress both mutant and wild-type *pfatp4*. What effect the expression of the transgene might have on expression/function of the native gene/protein (and whether, in particular, expression of the transgene decreases the expression of the native gene, as has been observed in at least one previous case [Sá et al., 2006]) is unknown. In addition, other compensatory mutations that were selected for in the direct drug-selected line could contribute to the spiroindolone-resistance phenotype.

The resting [Na^+^]_i_ in any cell results from the relative rates of Na^+^ efflux and influx (i.e., the so-called pump-leak balance). An increase in [Na^+^]_i_, as was seen here in response to a number of maneuvers (including the addition of spiroindolones), may thus arise as a consequence of either decreased Na^+^ efflux (pump) or increased Na^+^ influx (leak). The finding that NITD246 inhibits a membrane-associated Na^+^-dependent ATPase activity in infected (but not uninfected) erythrocytes provides direct evidence for the inhibition of a Na^+^ efflux pump. The findings that the spiroindolone causes the transmembrane pH gradient to increase and leaves [Ca^2+^]_i_ unperturbed argue against the compound inducing significant membrane leakage. Also, the finding that the rate of increase of [Na^+^]_i_ seen upon addition of maximally effective concentrations of the three most active spiroindolones was similar in each case, and similar to that seen on addition of orthovanadate (500 μM), is consistent with the time-dependent rise in [Na^+^]_i_, reflecting in each case the influx of Na^+^ via endogenous Na^+^ leak pathways, revealed by inhibition of the efflux pump. The identity of these influx pathways is unknown, though at least one Na^+^ influx transporter has been characterized previously in the intraerythrocytic parasite (Saliba et al., 2006).

The increase in pH_i_ seen on addition of NITD246 (or orthovanadate) to parasites suspended in the presence (but not in the absence) of extracellular Na^+^ might be explained if the Na^+^-ATPase extrudes Na^+^ in exchange for H^+^ (Figure 6). The extrusion of one cation in exchange for another is a common, and perhaps general, feature of cation-pumping P-type ATPases (Niggli and Sigel, 2008), and it has been proposed previously that the efflux of Na^+^ via ENA Na^+^-ATPases is accompanied by the countertransport of H^+^ (Rodríguez-Navarro and Benito, 2010). The influx of H^+^ into the parasite via the Na^+^-ATPase would constitute a significant acid load, which would be countered by the H^+^-extruding V-type H^+^-ATPase. Upon inhibition of the Na^+^-ATPase by either NITD246 or orthovanadate, the acid load is eliminated, shifting the balance between the influx of H^+^ (via the Na^+^-ATPase) and the efflux of H^+^ (via the H^+^-ATPase), with the ongoing extrusion of H^+^ via the V-type H^+^-ATPase resulting in an alkalinisation (inhibitable by the V-type H^+^-ATPase inhibitor concanamycin A). In cells washed and resuspended in Na^+^ free medium, [Na^+^]_i_ is reduced to close to zero, the Na^+^-ATPase no longer functions, and there is therefore no Na^+^-ATPase-associated acid load and no alkalinisation on inhibition of the Na^+^-ATPase.

The mechanism underpinning the increase in [Na^+^]_i_ seen on removal of K^+^ from the extracellular medium is unknown. Once again, the question arises of whether this increase is due to decreased Na^+^ efflux or increased Na^+^ influx. The observation that the rate of increase of [Na^+^]_i_ was 10-fold lower than the rate of increase of [Na^+^]_i_ following the addition of maximally-effective concentrations of inhibitors is consistent with the increase in [Na^+^]_i_ seen on removal of K^+^ not being due to the Na^+^-ATPase ceasing to operate under these conditions. If removal of extracellular K^+^ simply prevented the Na^+^-ATPase from extruding Na^+^, the rate of increase of [Na^+^]_i_ might be expected to be the same as that seen in response to pharmacological inhibition of the Na^+^-ATPase. Instead, these data are consistent with the removal of extracellular K^+^ causing an increase in Na^+^ influx, with the Na^+^-ATPase activity increasing as the parasite attempted to counter the rising [Na^+^]_i_. An increase in Na^+^-ATPase activity would result in increased H^+^ influx (via the countertransport mechanism), which would account for the progressive acidification of the cytosol seen for parasites suspended in a K^+^ free medium. The mechanism by which removal of K^+^ might induce increased Na^+^ influx was not investigated here.

The findings that spiroindolone-resistant parasites with mutations in PfATP4 showed increased resting [Na^+^]_i_, decreased Na^+^ efflux following an intracellular Na^+^ load, and increase sensitivity to the growth-inhibitory effects of excessive extracellular Na^+^ imply that there is some impairment of Na^+^ extrusion in these cells. Although it remains to be demonstrated directly that the spiroindolones bind to PfATP4, the data are consistent with them doing so and with the hypothesis that the resistance-conferring mutations alter the structure of the protein in such a way as both to reduce the affinity with which the spiroindolones bind and to compromise the protein’s ability to efflux Na^+^.

The extrusion of Na^+^, and thus the maintenance of a low [Na^+^]_i_, is a fundamental property of all nucleated cells. It is therefore likely that the parasite’s putative plasma membrane Na^+^-ATPase plays an essential housekeeping role. The observation that [Na^+^]_i_ increased significantly within a few minutes of inhibition of Na^+^ extrusion highlights the fact that the intraerythrocytic malaria parasite has a substantial Na^+^ influx, which, under normal conditions, is countered by Na^+^ efflux via the Na^+^-ATPase. The active efflux of Na^+^ represents a significant energy investment by the parasite, which not only uses ATP to expel Na^+^ (via the Na^+^-ATPase), but uses additional ATP (via the V-type H^+^-ATPase) to counter the acid load associated with the operation of the Na^+^-ATPase. What purpose (if any) might be served by the high endogenous influx of Na^+^ into the parasite and the consequent requirement for a high expenditure of ATP in maintaining a low [Na^+^]_i_ is unclear. Nevertheless, the high Na^+^ influx/efflux rate might be expected to make the parasite particularly vulnerable to chemical agents that interfere with the mechanisms involved.

## Experimental Procedures

### Parasite Culture, Isolation, and Growth Assays

*P. falciparum* strains 3D7, Dd2, NITD609-R^Dd2^ clone #2, Dd2^attB^, and Dd2^attB^ CAM I398F/P990R were cultured under shaking conditions as described elsewhere (Allen and Kirk, 2010). Cultures were synchronized 24 hr before experimentation using 5% w/v sorbitol (Lambros and Vanderberg, 1979). All experiments were conducted on mature trophozoite-stage parasites (36–40 hr postinvasion) functionally isolated from their host erythrocytes by permeabilization of the erythrocyte and parasitophorous vacuole membranes by brief exposure to saponin (0.05% w/v) as described previously (Spillman et al., 2008). After saponin isolation, parasites remained intact and able to generate and maintain transmembrane ion gradients (Saliba and Kirk, 1999; Spillman et al., 2008) as well as maintain a large inwardly negative membrane potential (Allen and Kirk, 2004).

Parasite growth assays were performed in 96-well plates over 48 hr (commencing at the ring stage) using a standard [^3^H]hypoxanthine incorporation assay (Desjardins et al., 1979).

### Inhibitors and Solutions

The four spiroindolones were synthesized as described previously (Yeung et al., 2010). All inhibitor stock solutions were prepared in dimethylsulfoxide, except those for orthovanadate and ouabain, which were dissolved in standard saline (125 mM NaCl, 5 mM KCl, 1 mM MgCl_2_, 20 mM glucose, 25 mM HEPES [pH 7.1]).

### Determination of [Na^+^]_i_, pH_i_, [Ca^2+^]_i_, and ATP

The [Na^+^]_i_ of saponin-isolated parasites was measured at 37°C using the Na^+^-sensitive dye SBFI (Molecular Probes, Invitrogen) in conjunction with a PerkinElmer LS 50B Fluorescence Spectrometer fitted with a Dual Excitation Fast Filter. Saponin-isolated parasites were loaded with SBFI by suspension (at 1.40–1.80 × 10^8^ cells/mL) for 20 min at 37°C in bicarbonate-free RPMI1640 supplemented with 20 mM D-glucose, 0.2 mM hypoxanthine, 25 mM HEPES, and 25 mg/L gentamycin sulfate (pH 7.10), to which SBFI-acetoxymethyl ester (5.5 μM) and Pluronic F-127 (0.01% w/v) were added. The dye-loaded cells were washed twice (12,000 × *g*, 0.5 min) in bicarbonate-free RPMI then incubated for a further 20 min at 37°C to allow for complete de-esterification of the dye before being resuspended at a final cell concentration of 1.5–2.5 × 10^7^ cells/mL in standard saline. The dye-loaded cells were excited at 340 nm and 380 nm with fluorescence recorded at 490 nm. Calibration of the relationship between the 340/380 nm fluorescence ratio and [Na^+^]_i_ (see Figures S1A–S1C) was carried out as described previously (Diarra et al., 2001; Harootunian et al., 1989). Several inhibitors used in this study caused inner filter effects (Gu and Kenny, 2009; Srinivas and Mutharasan, 1987); in these cases calibration curves were generated with the inhibitors present.

pH_i_ of saponin-isolated parasites was measured using the pH-sensitive indicator BCECF (Molecular Probes, Invitrogen) as described previously (Saliba and Kirk, 1999). The fluorescence from SBFI-loaded parasites varied significantly with pH_i_ (Figure S1C). Critical experiments performed using SBFI-loaded parasites were repeated with parasites loaded with BCECF. In the cases in which pH_i_ was found to vary during the course of the experiment, the SBFI fluorescence was corrected as described elsewhere (Diarra et al., 2001).

[Ca^2+^]_i_ was measured with the Ca^2+^-sensitive indicator fura-2 (Molecular Probes, Invitrogen) using a loading protocol identical to that described for SBFI. Calibration was performed as described previously (Alleva and Kirk, 2001).

ATP levels in isolated parasites were measured using firefly luciferase as described elsewhere (Saliba and Kirk, 1999).

### Membrane Preparation and ATPase Assays

For the preparation of uninfected erythrocyte membranes, packed uninfected erythrocytes were lysed by incubation for 5 min in ice-cold PBS containing 0.1% (w/v) saponin together with 1/500 Protease Inhibitor Cocktail Set III (Calbiochem). The erythrocyte ghosts were pelleted by centrifugation (12,000 × *g*, 10 min) and washed three times in ice-cold water (12,000 × *g*, 10 min) immediately prior to their use in the ATPase assay. To prepare membranes from parasitised erythrocytes, saponin-isolated parasites were lysed by suspension in ice-cold water (containing 1/500 Protease Inhibitor Cocktail Set III) then washed three times in ice-cold water (12,000 × *g*, 10 min) before their immediate use in the ATPase assay. The ATPase activity of membrane preparations from uninfected erythrocytes and saponin-isolated parasites was estimated from the rate of hydrolysis of ATP and measured using the PiColorLock Gold Phosphate (P_i_) Detection System (Innova Biosciences). Briefly, membrane preparations from 1–3 × 10^8^ cells were suspended in reaction buffer (100 mM of either NaCl or choline chloride, 50 mM Tris-HCl, 20 mM KCl, and 2 mM MgCl_2_) at 37°C. The ATPase reaction was initiated by the addition of 0.25 mM Na_2_ATP. Note that it was necessary to use the Na^+^ salt of ATP, rather than the K^+^ and Mg^2+^ salts, as the latter both contain residual P_i_ that saturated the P_i_ detection system. As a result, the low-Na^+^ solution used in the ATPase assays contained 0.5 mM Na^+^. At the required time points (typically every 5–10 min over a 20 min time course), triplicate aliquots of the reaction mixture were transferred to a 96-well plate containing the “gold mix,” which terminated the ATPase reaction. Further sample processing was performed as per the kit instructions.

### Data Analysis

In experiments in which inhibitors were shown to cause a time-dependent rise in [Na^+^]_i_, the initial Na^+^ influx rate was estimated by fitting the following function to the data: [Na^+^]_i_ = [Na^+^]_i_^t = 0^ + Δ[Na^+^]_i_^max^ × (1-e^-*a*t^), where [Na^+^]_i_^t = 0^ is the initial resting [Na^+^]_i_, Δ[Na^+^]_i_^max^ is the maximum increase in [Na^+^]_i_, t is the time after the addition of inhibitor, and *a* is a fitted constant. The initial Na^+^ influx rate (at t = 0) is a × Δ[Na^+^]_i_^max^.

The rate of efflux of Na^+^ from the cells, following an imposed intracellular Na^+^ load, was estimated by fitting the following function to the [Na^+^]_i_ traces: [Na^+^]_i_ = Δ[Na^+^]_i_^max^ × e^-*a*t^ + [Na^+^]_i_^final^, where Δ[Na^+^]_i_^max^ is the increase in [Na^+^]_i_ above the normal resting value (following the imposition of an intracellular Na^+^-load), t is the time after the commencement of efflux (on addition of extracellular K^+^), *a* is a fitted constant, and [Na^+^]_i_^final^ is the final value of [Na^+^]_i_. The initial Na^+^ efflux rate (at t = 0) is *a* × Δ[Na^+^]_i_^max^, and the half-time to complete recovery (t_1/2_) is ln(0.5)/-*a*.

Dose-response curves were obtained using the expression y = y^min^ + y^max^/(1 + [C/IC_50_]^b^), where y is the parameter being measured (parasite proliferation, Na^+^ influx, or ATPase activity), y^min^ is the minimum value of y, y^max^ is the maximum value of y, C is the concentration of the moiety that was varied in the experiment, IC_50_ is the concentration at which y was reduced to 50% of y^max^, and b is a fitted constant.

Statistical comparisons were made using an unpaired t test unless stated otherwise.

## Figures and Tables

**Table 1:** Characterization of PfATP4 Mutant Parasites and their Parent Lines in Terms of [Na^+^]_i_ Regulation and their Sensitivity to Spiroindolones

Strain	Resting [Na^+^]_i_ (mM)	Half-time for recovery of [Na^+^]_i_ following Na^+^ loading (s)	IC_50_ for inhibition of parasite proliferation by excess extracellular Na^+^ (mM)	IC_50_ for inhibition of parasite proliferation by NITD246 (nM)	IC_50_ for disruption of Na^+^ regulation by NITD246 (nM)	IC_50_ for disruption of ATPase activity by NITD246 (nM)
Dd2 parent	7.2 ± 1.1 (10)	153 ± 22 (5)	58 ± 5 (5)	0.08 ± 0.01 (5)	1.6 ± 0.3	0.15 ± 0.05
NITD609-R^Dd2^ clone #2	14.8 ± 1.9 (10)^∗^	345 ± 10 (4)^∗^	31 ± 2 (7)^∗^	0.89 ± 0.12 (7)^∗^	10.4 ± 8.4	1.1 ± 0.2
Dd2^attB^ parent	8.4 ± 0.7 (11)	158 ± 7 (4)	49 ± 8 (5)	0.08 ± 0.01 (5)	1.7 ± 0.5	0.06 ± 0.01
Dd2^attB^ CAM I398F/P990R	11.9 ± 1.8 (8)	335 ± 55 (4)^∗^	32 ± 5 (5)	0.61 ± 0.14 (5)^∗^	3.9 ± 1.7	0.17 ± 0.12

IC_50_ values for inhibition of parasite proliferation were estimated using a standard [^3^H]-hypoxanthine incorporation assay In the case of inhibition of parasite proliferation by excess Na^+^ the IC_50_ values represent the concentrations of excess Na^+^ (i.e., the increase in [Na^+^] above that in standard RPMI) required to inhibit proliferation by 50%. Resting [Na^+^]_i_ was determined in SBFI-loaded saponin-isolated trophozoites suspended in standard saline. The half-time for recovery from an imposed intracellular Na^+^ load was calculated by fitting an exponential decay function to the time-course for recovery seen on addition of 10 mM KCl to cells preloaded to a [Na^+^]_i_ approximately double the normal resting value by suspension in a K^+^-free medium (as in Figure 3A). The IC_50_ values for disruption of Na^+^ regulation are the concentration of each inhibitor required to cause the [Na^+^]_i_ to increase from its normal resting value at half the maximal rate. The IC_50_ values for disruption of ATPase activity by NITD246 are the concentration of each inhibitor required to cause the P_i_ production rate to decrease by half the maximal amount. For all columns other than those pertaining to the disruption of [Na^+^]_i_ regulation and the disruption of ATPase activity, the IC_50_ values cited are, in each case, the mean ± SEM of those estimated in the number of independent experiments shown in parentheses (with each independent experiment performed in triplicate). The IC_50_ values for disruption of [Na^+^]_i_ regulation and ATPase activity were estimated from curves fitted to data such as those shown in Figure 5, with each data point representing the mean of at least three independent experiments. Asterisks indicate a statistically significant difference between the parent line and the parasites expressing mutant PfATP4 (p < 0.05).
