# Supplementary material for: Na+ Regulation in the Malaria Parasite Plasmodiumfalciparum Involves the Cation ATPase PfATP4 and Is a Target of the Spiroindolone Antimalarials
Source: Cell Host Microbe. 2013 Feb 13;13(2):227–37. doi: 10.1016/j.chom.2012.12.006 (PMC3574224; doi:10.1016/j.chom.2012.12.006)
Supplement: Document S1. Figures S1 and S2 [file mmc1.pdf]

Cell Host & Microbe, Volume *13*

Supplemental Information

Na^+ Regulation in the Malaria Parasite *Plasmodium falciparum* Involves the Cation ATPase PfATP4 and Is a Target of the Spiroindolone Antimalarials

Natalie J. Spillman, Richard J. W. Allen, Case W. McNamara, Bryan K. S. Yeung, Elizabeth A. Winzeler, Thierry T. Diagana, and Kiaran Kirk

Supplemental Figure 1

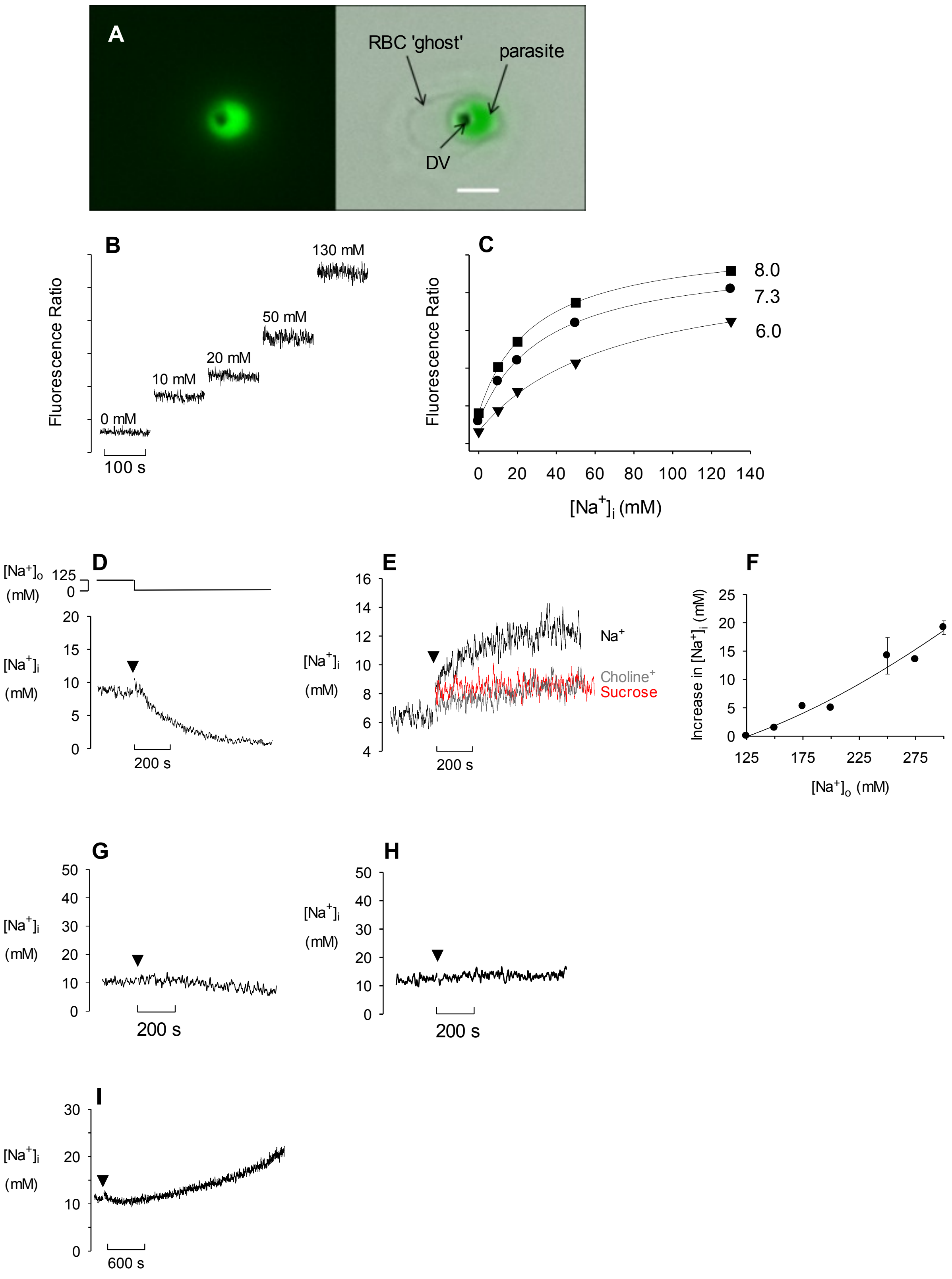

Figure S1. Na^+ Regulation in Saponin-Isolated *P. falciparum* Trophozoites, Related to Figure 1

(A) Localisation of SBFI fluorescence in a wet-mount preparation of saponin-isolated trophozoites observed using epifluorescence microscopy (FView II digital camera mounted on an Olympus IX81 inverted epifluorescence microscope fitted with a 100x (1.4 NA) objective lens and DAPI filter cube (340-380 nm excitation, 425 nm long pass emission)). The left panel is the fluorescence image and the right panel is the fluorescence image superimposed on the brightfield image. Image analysis was performed using NIH ImageJ (version 1.40; http://rsb.info.nih.gov/ij/). Fluorescence was observed from the parasite cytoplasm, but absent from the erythrocyte ghost and digestive vacuole (DV) compartments. The scale bar is ~3 µm.

(B) Representative calibration traces for SBFI-loaded parasites equilibrated to varying $[Na^+]_i$ using a combination of the ionophores nigericin (5 µM), gramicidin (2.5 µM) and monensin (5 µM). Calibration solutions, containing 0, 10, 20, 50 or 130 mM Na^+, were generated by mixing solutions containing 80 mM Na^+/K^+ gluconate and 50 mM NaCl/KCl (thereby ensuring $[Cl^-]_o \approx [Cl^-]_i$ (Henry et al., 2010)), 1 mM $MgCl_2$, 20 mM glucose and 25 mM HEPES). A five-point calibration was performed for every experiment, and a hyperbolic standard curve was generated as described previously (Diarra et al., 2001; Grynkiewicz et al., 1985). The curves are representative of those obtained in at least five independent experiments.

(C) Representative calibration curves showing the relationship between fluorescence ratio and $[Na^+]_i$ at three different pH values: 6.0, 7.3 and 8.0. The data obtained at each pH were fit to a hyperbolic function ($R^2 > 0.95$) which was used subsequently to convert the measured fluorescence ratio to $[Na^+]_i$ using the methods described by Diarra and colleagues (Diarra et al., 2001). The curves are representative of those obtained in at least five independent experiments.

(D) Effect of removal of extracellular Na^+ on $[Na^+]_i$. Extracellular Na^+ was replaced with choline$^+$ at the timepoint indicated by the closed triangle. The extracellular Na^+ concentration over the course of the experiment is shown above the trace. The trace is representative of that obtained in at least three independent cell preparations.

(E) Effect of increasing extracellular $[Na^+]$ on $[Na^+]_i$. Extracellular $[Na^+]$ ($[Na^+]_o$) was increased (above the value of 125 mM in standard saline) by the addition of 75 mM NaCl at the timepoint indicated by the closed triangle (black trace). Isosmotic quantities of either choline chloride (grey trace) or sucrose (red trace) were added to separate cell aliquots to allow an assessment of the extent to which $[Na^+]_i$ increased as a result of osmotic shrinkage of the parasite. The traces are representative of those obtained in at least five independent cell preparations.

(F) Dependence of $[Na^+]_i$ on $[Na^+]_o$ as $[Na^+]_o$ was increased above 125 mM. $[Na^+]_i$ was determined 10 min after the increase in $[Na^+]_o$. At each $[Na^+]_o$, the increase in $[Na^+]_i$ measured following the addition of an equimolar concentration choline chloride was subtracted from that measured following addition of NaCl, in order to correct for the increase in concentration arising from osmotic shrinkage of the parasite and to provide, thereby, an estimate of the magnitude of the 'shrinkage-independent' increase in $[Na^+]_i$. Where not shown the error bars lie within the data point. The line was drawn using a sigmoidal curve fitted to the data ($R^2 = 0.98$). Each data point is averaged from between three and five independent experiments (mean ± S.E.M.).

(G)(H) Effect of the antimalarials (G) chloroquine (10 µM) and (H) artemisinin (10 µM) on $[Na^+]_i$. The drugs were added at the time-point indicated by the closed triangle. The traces shown are, in each case, representative of those obtained from at least three independent cell preparations.

(I) Effect of glucose removal (and hence ATP depletion) on $[Na^+]_i$. The parasites were washed and then resuspended in glucose-free saline at the point indicated by the closed triangle. The trace is representative of those obtained in four independent experiments.

Supplemental Figure 2

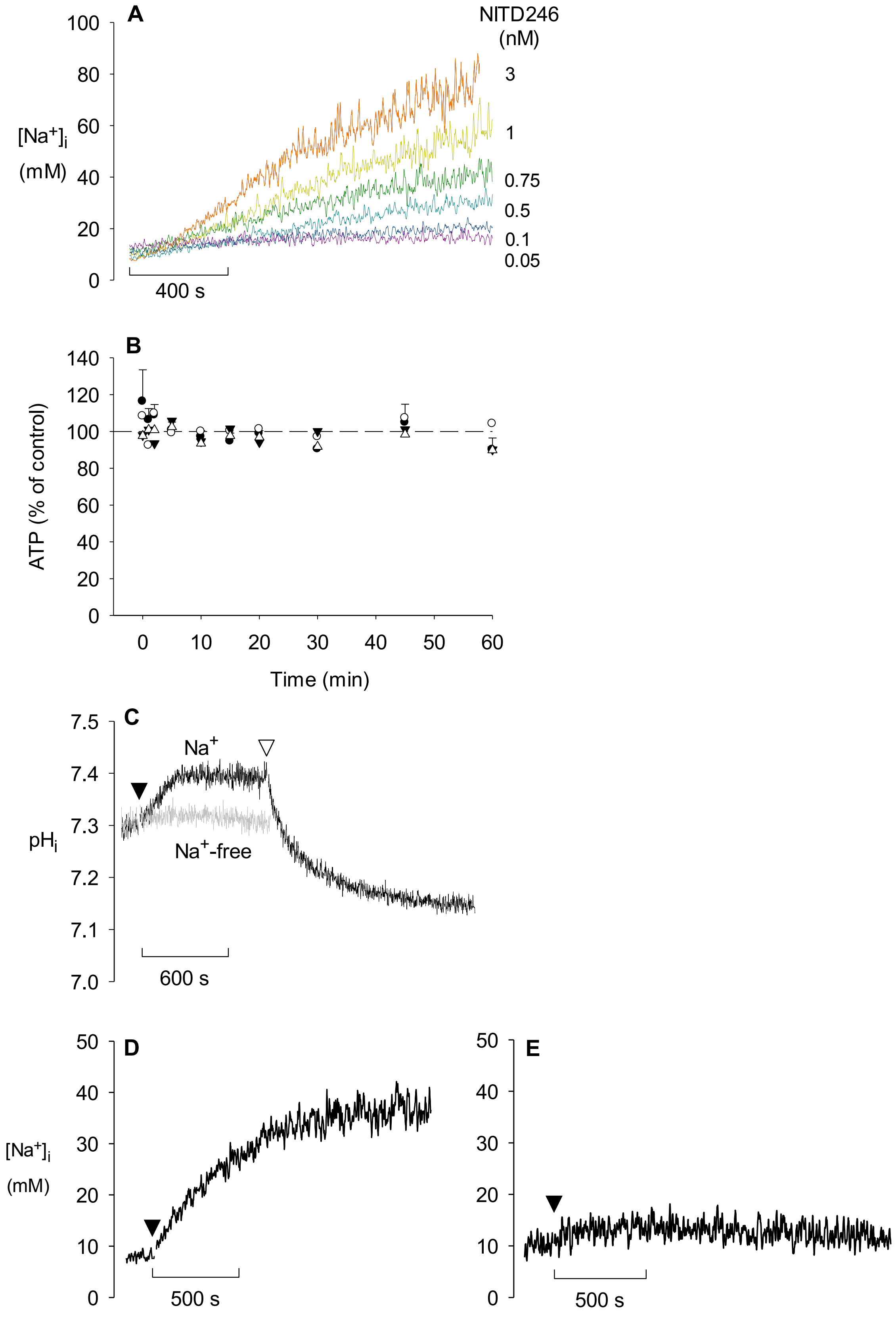

Figure S2. Effects of Spiroindolones and Other Inhibitors on Ion Regulation in Saponin-Isolated *P. falciparum* Trophozoites, Related to Figure 2

(A) Concentration-dependent disruption of $[Na^+]_i$ regulation in SBFI-loaded, saponin-isolated *P. falciparum* trophozoites by the spiroindolone NITD246. The spiroindolone was added at the start of each trace. The traces shown are representative of those obtained from at least three independent cell preparations.

(B) Lack of effect of the spiroindolones on ATP levels in saponin-isolated parasites. Intracellular [ATP] was determined using the firefly luciferase assay and results were standardised to the resting cytosolic [ATP] under glucose-containing, inhibitor-free conditions (unchanged across the timecourse; data not shown). Inhibitors (● NITD246; ○ NITD247; ▼ NITD138; △ NITD139) were added at time-zero. The data are averaged from three independent experiments (mean ± S.E.M.). For clarity, error bars are shown for the NITD246 series only, and where not shown error bars lie within the symbol. The dashed reference line indicates 100 % ATP (control levels).

(C) Effect on pH_i of the addition of orthovanadate (100 µM, at the point indicated by the closed triangle) followed by the addition of concanamycin A (75 nM, at the point indicated by the open triangle) to BCECF-loaded parasites suspended in either standard saline (black trace) or Na^+-free solution (in which Na^+ was replaced with an equimolar concentration of choline$^+$; grey trace). The addition of orthovanadate induced an alkalinisation in the presence, but not in the absence, of extracellular Na^+. On addition of the V-type H^+-ATPase inhibitor concanamycin A to parasites in Na^+-containing medium that had undergone an NITD246-induced alkalinisation there was an immediate reversal of the alkalinisation, with pH_i decreasing to below its normal resting value and approaching pH_o. The traces shown are representative of those obtained from at least three independent cell preparations.

(D)(E) Effect of (D) cyclopiazonic acid (40 µM) and (E) thapsigargin (2 µM) on $[Na^+]_i$ in SBFI-loaded, saponin-isolated *P. falciparum* trophozoites. Addition of each inhibitor is indicated by a closed triangle. The traces shown are representative of those obtained from at least three independent cell preparations.

Supplemental References

Diarra, A., Sheldon, C., and Church, J. (2001). *In situ* calibration and [H^+] sensitivity of the fluorescent Na^+ indicator SBFI. Am J Physiol Cell Physiol *280*, C1623-1633.

Grynkiewicz, G., Poenie, M., and Tsien, R.Y. (1985). A new generation of Ca^{2+} indicators with greatly improved fluorescence properties. J Biol Chem *260*, 3440-3450.

Henry, R.I., Cobbold, S.A., Allen, R.J., Khan, A., Hayward, R., Lehane, A.M., Bray, P.G., Howitt, S.M., Biagini, G.A., Saliba, K.J.*, et al.* (2010). An acid-loading chloride transport pathway in the intraerythrocytic malaria parasite, *Plasmodium falciparum*. J Biol Chem *285*, 18615-18626.
